# COSMOS: cloud enabled NGS analysis

**DOI:** 10.1186/1471-2105-16-S2-A2

**Published:** 2015-01-28

**Authors:** Yassine Souilmi, Jae-Yoon Jung, Alex Lancaster, Erik Gafni, Saaid Amzazi, Hassan Ghazal, Dennis Wall, Peter Tonellato

**Affiliations:** 1 Department of Biology, Faculty of Sciences of Rabat, Morocco; 2 Center for Biomedical Informatics, Harvard Medical School, Boston, MA 02115, USA; 3 INVITAE, San Francisco, CA 94107, USA; 4 Department of Biology, Mohamed First University, Oujda/Nador, Morocco; 5 Department of Pediatrics, Division of Systems Medicine, Stanford University, Stanford, CA 94305, USA

## Background

The dramatic fall of next generation sequencing (NGS) cost in recent years positions the price in range of typical medical testing, and thus whole genome analysis (WGA) may be a viable clinical diagnostic tool. Modern sequencing platforms routinely generate petabyte data. The current challenge lies in calling and analyzing this large-scale data, which has become the new time and cost rate-limiting step.

## Methods

To address the computational limitations and optimize the cost, we have developed COSMOS (http://cosmos.hms.harvard.edu) , a scalable, parallelizable workflow management system running on clouds (e.g., Amazon Web Services or Google Clouds). Using COSMOS [1], we have constructed a NGS analysis pipeline implementing the Genome Analysis Toolkit - GATK v3.1 - best practice protocol [2,3], a widely accepted industry standard developed by the Broad Institute. COSMOS performs a thorough sequence analysis, including quality control, alignment, variant calling and an unprecedented level of annotation using a custom extension of ANNOVAR. COSMOS takes advantage of parallelization and the resources of a high-performance compute cluster, either local or in the cloud, to process datasets of up to the petabyte scale, which is becoming standard in NGS.

## Conclusion

This approach enables the timely and cost-effective implementation of NGS analysis, allowing for it to be used in a clinical setting and translational medicine. With COSMOS we reduced the whole genome data analysis cost under the $100 barrier, placing it within a reimbursable cost point and in *clinical time*, providing a significant change to the landscape of genomic analysis and cement the utility of cloud environment as a resource for Petabyte-scale genomic research.
